# The Lin28 Expression in Stallion Testes

**DOI:** 10.1371/journal.pone.0165011

**Published:** 2016-10-31

**Authors:** Geumhui Lee, Heejun Jung, Minjung Yoon

**Affiliations:** 1 Department of Horse, Companion, and Wild Animal Science, Kyungpook National University, Sangju, Republic of Korea; 2 Department of Animal Science and Biotechnology, Kyungpook National University, Sangju, Republic of Korea; Centre de Recherche en Cancerologie de Lyon, FRANCE

## Abstract

The molecular markers for specific germ cell stages can be utilized for identifying, monitoring, and separating a particular stage of germ cells. The RNA-binding protein Lin28 is expressed in gonocytes of human fetal testes. The Lin28 expression is restricted to a very small population of spermatogonial cells in human, mice, and monkey. The main objective of this study was to investigate the expression pattern of Lin28 in stallion testes at different reproductive stages. Based on the presence or absence of full spermatogenesis and lumina in seminiferous tubules, the testicular samples were categorized into two reproductive stages pre-pubertal and post-pubertal. We performed a reverse transcription polymerase chain reaction to confirm the presence of Lin28 mRNA in the testicular tissues and a western blot analysis to verify the cross-reactivity of rabbit Lin28 antibody with horse testicular tissue. For immunohistochemistry, Lin28 (rabbit anti-human), GATA4 (goat anti-human) or DAZL (goat anti-human) antibodies were used. The results of RT-PCR confirmed the expression of Lin28 mRNA in the stallion testes. The western blot analysis showed that the expression of 28 kDa Lin28 protein was localized in the cytoplasm of spermatogonia at both reproductive stages. The numbers of Lin28-positive germ cells per 1000 Sertoli cells in pre- and post-pubertal stages were 253 ± 8.66 and 29.67 ± 2.18, respectively. At both reproductive stages, all Lin28 positive cells showed no co-stained with GATA4 antibody, whereas only some of the Lin28-positive germ cells showed co-staining with DAZL antibody. The results from whole-mount staining showed that the Lin28 expression was limited to A_single_ (A_s)_ and A_paired_ (A_pr_) spermatogonia. In conclusion, Lin28 might be utilized as a molecular marker for undifferentiated spermatogonial stem cells when used with DAZL antibody.

## Introduction

Spermatogonial stem cells (SSCs) have the potential to undergo self-renewal and differentiation for continuous sperm production, and therefore can be used as a resource to preserve the genetic value of stallions. The formation of spermatogonial colonies in the seminiferous tubules of infertile recipients after transplantation of SSCs is considered a sole biomarker for identification of SSCs [1]. Besides, the utilization of putative molecular markers for undifferentiated SSCs has been introduced as an alternative method to identify certain developmental stages of SSCs [2]. In stallions, GFRα1, PLZF, and CSF1R have been identified as markers for undifferentiated spermatogonia [3]. However, the molecular markers specific for different stages of spermatogonia have not been identified because whole-mount staining is not feasible with these markers. Previously, we have reported that UTF1 is a molecular marker for undifferentiated type A spermatogonia [4]. However, the UTF1 protein was found to be expressed in A_single_ (A_s_)_,_ A_paired_ (A_pr_), and chains of 4, 8, and 16 A_aligned_ (A_al_) types of spermatogonia. Some studies have suggested that the chains of 4–16 A_al_ spermatogonia are generally known to be differentiated [2], whereas others have argued that the chains of 4 A_al_ spermatogonia or beyond also contain stem cell potential [5]. Although the most advanced stage of spermatogonia for undifferentiated SSCs is unclear, it is certain that the less advanced stage of spermatogonia are more likely to be undifferentiated. Thus, we sought to identify another putative molecular marker for a stage of spermatogonia earlier than 16 A_al_.

Lin28 is a protein encoded by the *LIN28* gene [6]. It inhibits the processing of microRNAs (miRNAs) into mature miRNAs by binding to the terminal loops of miRNA precursors such as let-7 family members [6]. Thus, Lin28 is suggested to play a role in blocking miRNA-mediated differentiation of stem cells and certain cancers [7]. The expression of Lin28 in testicular cells was first demonstrated in the undifferentiated spermatogonia (A_s_ to A_al_) of adult mice [8]. In marmoset monkey, the expression of Lin28 was found in the primordial germ cells during the prenatal period and in a few germ cells in all reproductive stages [9, 10]. Lin28 expression has also been reported in a rare population of adult human spermatogonia [10]. These findings suggest that Lin28 might be utilized as a molecular marker for undifferentiated SSCs in stallions.

The main objectives of this study were 1) to confirm the expression of Lin28 protein in the stallion testis at different reproductive stages and 2) to identify the subpopulation of Lin28-positive spermatogonia. Based on the evidences from previous studies on other species, we hypothesize that Lin28 is a putative marker for stallion SSCs.

## Materials and Methods

### 1. Animals

Testicular samples were collected from light-horse breeds including Thoroughbred and Jeju horses through a routine field castration performed at private horse farms in the Republic of Korea S1 Table. Based on the age and presence of lumina in the cross-sections of the seminiferous tubules of stallions, their reproductive stages were categorized as follows: pre-pubertal (< 1.5 yr, n = 3) and post-pubertal (2–4 yr, n = 3) [4, 11].

### 2. Testicular tissue sample preparation

Preparation of testicular tissue samples was performed as previously described [4] with slight modifications. Briefly, after castration, the testes were transported to the laboratory in an icedbox maintained at 4°C. The fixation process was performed by immersing the testicular parenchyma (1 cm^3^) in 4% paraformaldehyde (Duksan, Ansan-si, Gyeonggi-do, Korea) for at least 24 h at room temperature. Subsequently, the tissues were dehydrated using a series of ethanol concentrations, and then embedded in paraffin. For western blotting, pieces of testicular tissue (0.5 cm^3^) were snap-frozen in liquid nitrogen and stored at −80°C until use.

### 3. Molecular analysis

Testicular tissues of stallions were homogenized to extract total RNA using a Polytron PT 1200 CL (Kinematica AG, Littau-Lucerne, Switzerland). The extracted total RNA was quantified and diluted to a concentration of 100 ng/μl for reverse transcription polymerase chain reaction (RT-PCR). The Lin28 primers, Fw. 5′-TGTAAGTGGTTCAACGTGCG-3′ and Rev. 5′-CAGCTTACTCTGGTGCACAA-3′ (Macrogen, Seoul, Rep. of Korea), were diluted to a final concentration of 100 mM with dH2O (TaKaRa, Seoul, Rep. of Korea). RT-PCR was performed using a Thermal Cycler (BIO RAD, Hercules, CA, USA) with the following cycling conditions: incubation at 50°C for 30 min; activation at 95°C for 15 min; 40 cycles of denaturation at 95°C for 30 s, annealing at 60°C for 30 s, and extension at 72°C for 1 min; and a final extension at 72°C for 5 min. The samples were electrophoresced on a 2% agarose gel for 25 min, incubated in ethidium bromide reagent (BIONEER, Daejeon, Rep. of Korea) for 10 min, and then washed with distilled water for 10 min. The expression levels of Lin28 were analyzed with Biodoc-lt Imaging System (UVP, Upland, CA, USA) and captured by manual zoom camera (Computar, Cary, NC, USA).

### 4. Western blot analysis

Western blot analysis was performed to verify the cross-reactivity of rabbit anti-human Lin28 antibody to Lin28 protein in the equine testis as previously described [4] with minor modification. Briefly, for protein sample preparation, thawed samples were homogenized using a Polytron PT 1200 CL (Kinematica AG, Littau-Lucerne, Switzerland) in a radioimmunoprecipitation assay buffer for approximately 5 min. The protein concentration in ach sample was determined using Bradford Bio-Rad Total Protein assay (Bio-Rad Laboratories, Inc., Hercules, CA). The homogenized tissues were diluted in the sample preparation buffer [0.5 M Tris-HCL (pH 6.8), 0.1% glycerol (w/v), 10% sodium dodecyl sulfate (SDS; w/v), 0.05% 2-β-mercaptoethanol (w/v), and bromophenol blue in distilled water] to a concentration of 1 mg/mL. After heating the samples in a boiling water bath for 10 min, each protein sample (15 μL) was loaded onto a 10% SDS-polyacrylamide gel and electrophoresced using a Mini-Protean II system (Bio-Rad). Proteins were electro-transferred to a membrane (Millipore, Bedford, MA, USA) and blocked with the Blotto reagent (Santa Cruz Biotechnology, CA, USA). The membrane was then incubated with the Lin28 antibody (rabbit anti-human, AB63740, 1:500; Abcam, Cambridge, UK) diluted in the Blotto reagent overnight at 4°C. For the negative control, the membrane was treated with normal rabbit serum (Sigma, St. Louis, MO, USA) using the same immunoglobulin (IgG) concentration as that of the primary antibodies. For the secondary antibody, anti-rabbit IgG HRP (Cell Signaling, Boston, MA, USA) was used at a 1:10,000 dilution for 1 h at room temperature. The DEVELOPER (1:10, ILFORD, Cheshire, England) and HYPAM solution (1:5, ILFORD) were used to develop the photographic film in the dark room.

### 5. Immunofluorescence

Immunofluorescence was performed as previously described [11] with minor modification. Briefly, 5-μm sections of the testicular tissues were treated with xylene (Duksan) to remove the paraffin and then rehydrated in a graded series of ethanol washes. The antigen retrieval of testicular tissue was performed by incubating the tissues in citrate buffer at 97.5°C for 30 min and blocked with 5% donkey serum (Sigma) diluted in phosphate-buffered saline (PBS). The testicular tissues were incubated with rabbit anti-human Lin28 diluted 1:100 in blocking buffer for 1.5 h in a humid chamber. After washing thrice for 5 min, the testicular tissues were treated with the secondary antibody at a dilution of 1:1,000 (donkey anti-rabbit IgG Alexa Fluor 488, Life Technologies, Grand Island, NY, USA) for 45 min. The antibody-stained testicular tissues were mounted in Vectashield mounting medium containing 4, 6-diamidino-2-phenylindole (DAPI, Vector Laboratories, Burlingame, CA, USA). For counterstaining, goat anti-human GATA4 antibody (1:100, Santa Cruz Biotechnology) or goat anti-human DAZL (deleted in azoospermia-like) antibody (1:100, Sigma) were used as primary antibodies. As a secondary antibody for these counterstains, donkey anti-goat IgG Alexa Fluor 594 (1:1000, Life Technologies) was used.

### 6. Single germ cell isolation from testicular tissues for immunocytochemistry

For immunocytochemistry, single germ cells were isolated from testes in pre-pubertal (n = 3) and post-pubertal (n = 3) stallions using a two-step enzyme protocol as previously reported [4], with slight modifications. Briefly, the sliced testicular tissues (1 cm^3^) were incubated with collagenase type IV (1 mg/mL; Sigma) dissolved in Hank’s balanced salt solution (HBSS; Invitrogen) at 37°C for 15 min with vigorous shaking in a shaking incubator (Vision Scientific, Yuseong Gu, Daejeon, Korea). After centrifugation at 200 ×*g* for 10 min the supernatant containing Leydig cells was removed. Subsequently, the Leydig cells were treated with trypsin (2.0 mg/mL trypsin plus 1.04 mM EDTA; Invitrogen) and DNase I (1.4 mg/mL; Sigma) dissolved in HBSS for 15 min. The fetal bovine serum (FBS, 10%) was used to quench the digestion. The testicular cell solution was filtered through a 70-μm cell strainer (Becton Dickinson and Company, Franklin Lakes, NJ, USA) and centrifuged at 600 ×*g* for 10 min. Subsequently, the testicular cell pellets were resuspended in minimum essential medium α (MEMα) supplemented with 10% FBS. Approximately 5 × 10^4^ germ cells were fixed with ice-cold methanol and mounted onto the Fisherbrand^TM^ Superfrost/Plus microscope slides (Fisher Scientific, Fisher Scientific Company, Ottawa, Canada). The fixed germ cells were blocked with donkey serum and prepared for immunofluorescence using the procedure described above.

### 7. Whole-mount immunofluorescent staining of seminiferous tubules

After the first testicular digestion with collagenase, the dispersed seminiferous tubules were fixed in 4% paraformaldehyde overnight at 4°C. Subsequently, the tubules were washed with PBS thrice at 60-min intervals, and dehydrated in a series of 25%, 50%, 75%, 95%, and 100% methanol (MeOH) for 10 min. Thereafter, the seminiferous tubules were permeabilized with 3 mL of MeOH:DMSO:H_2_O_2_ (4:1:1) for 3 h. The tubules were re-hydrated in 3 mL of 50 and 25% MeOH in PBS for 10 min, and then washed in PBS twice for 15 min each.

The tubules were blocked (2 × 15 min, 1 × 1.5 h) in ice-cold PBSMT blocking buffer (2% Blotto milk powder and 0.5% Triton X-100 in PBS). The tubules were then incubated with the Lin28 antibody (1:300) diluted in blocking buffer at 4°C overnight. After washing with PBSMT (2 × 15 min, 5 × 1 h), the tubules were treated with donkey anti-rabbit IgG Alexa Fluor 488 (1:1,000; Life Technologies) diluted in blocking buffer at 4°C overnight. The tubules were mounted on the Fisherbrand^TM^ Superfrost/Plus microscope slides (Fisher Scientific) with Vectashield mounting medium containing DAPI (Vector Laboratories) after washing with ice-cold PBSMT (2 × 15 min, 5 × 1 h) followed by washing with PBS twice for 10 min each.

### 8. Imaging

The immunostained tissues were examined using a Leica DM 2500 fluorescent microscope (Leica, Wetzlar, Germany) equipped with an EL 6000 external light source (Leica), and images were captured using Leica DFC 450 C camera. Green and red fluorescent signals were observed using a dual-emission FITC/TRITC filter. The immunolabeling of single germ cells was observed using a confocal laser scanning microscope (Carl Zeiss, LSM 700) And the images were captured using a LSM T-PMT camera (Carl Zeiss). Cell counting was performed manually by a well-trained observer. Cells stained with green fluorescence were considered Lin28-positive, whereas the cells not stained with any color of fluorescence were considered Lin28-negative.

### 9. Statistical analysis

Several microscopic fields of testicular tissues stained with both Lin28 and GATA4 antibody were observed to count the population of Lin28-positive cells out of 1000 GATA4 positive cells for each animal sample by a blinded researcher S2 Table. The number of Lin28-positive germ cells per 1000 Sertoli cells in pre- (n = 3) and post-pubertal stallion testes (n = 3) were statistically compared using a *t*-test (SPSS V22). Various microscopic fields of testicular tissues stained with both Lin28 and DAZL antibodies were used to calculate the percentages of spermatogonia immunolabeled with only Lin28, both Lin28 and DAZL, or only DAZL. The percentage was determined by counting 2000 spermatogonia per animal. The spermatogonia were identified on the basis of both their morphological characteristics and their location in the cross-section of a seminiferous tubule. The percentage of spermatogonia immunolabeling in pre- (n = 3) and post-pubertal (n = 3) stallion testes were also statistically compared using a *t*-test (SPSS V22). The P-values < 0.05 were considered statistically significant.

## Results

### 1. Lin28 mRNA expression in the testicular tissue

**Fig 1:**
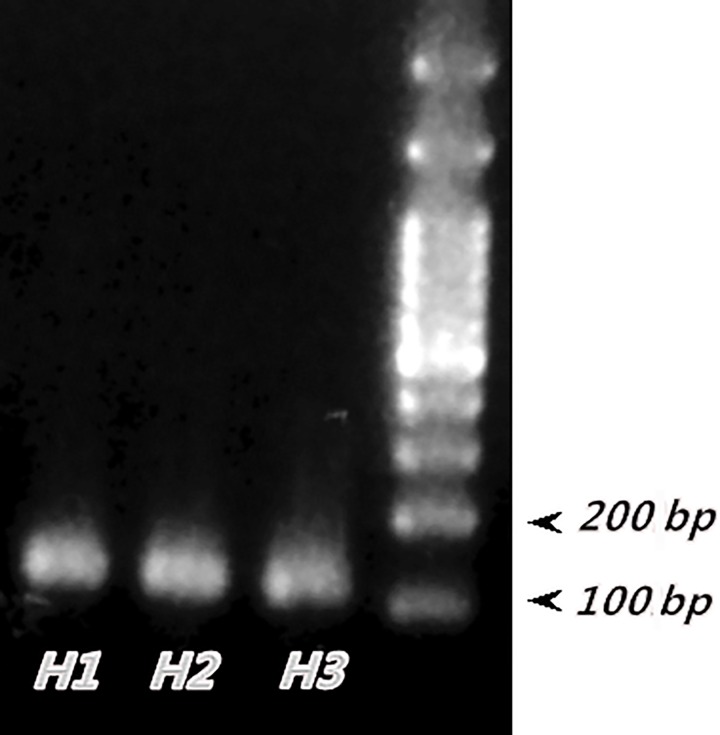
Expression of Lin28 mRNA in testes of stallions. The presence of Lin28 transcript in the testicular tissues of pre-pubertal stallions was demonstrated. The size of Lin28 mRNA was 108 bp. H = horse ID.

To test the expression of Lin28 protein in testes, the testicular tissues were collected from 3 pre-pubertal stallions and examined for mRNA expression using RT-PCR. The result of RT-PCR revealed that Lin28 mRNA was expressed in the testes of pre-pubertal stallion (Fig 1).

### 2. Cross-reactivity of Lin28 antibody in stallion testes

**Fig 2:**
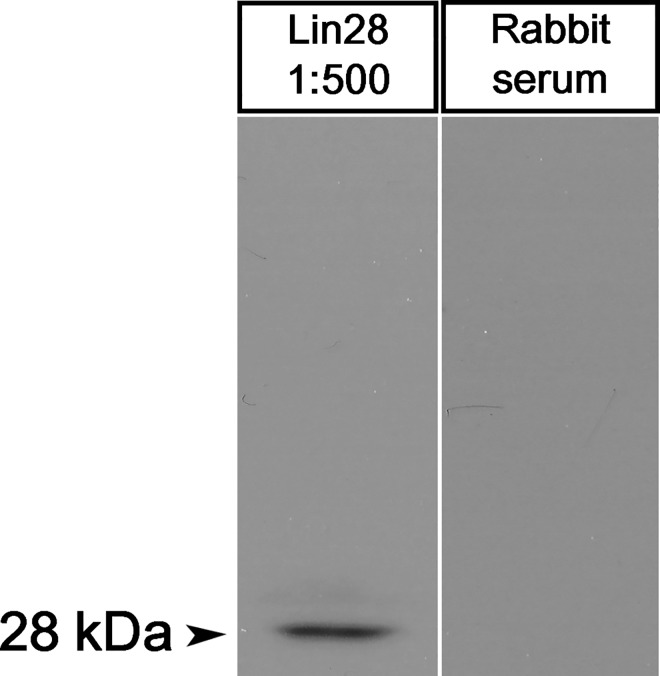
Cross-reactivity of Lin28 antibody in horse testes. The protein band of the Lin28 appeared at the molecular weight of 28kDa. The negative control lane was treated with rabbit serum with the same dilution as that of the primary antibody. No band was observed in the negative control lane unlike the positive control lane.

The cross-reactivity of Lin28 antibody to the testicular tissues of pre-pubertal stallions (n = 3) was assessed through western blotting. The protein band that reacted with Lin28 antibody appeared at an approximate molecular weight of 28 kDa (Fig 2) which is the known molecular weight of Lin28 [10]. The lane for negative control, which was treated with rabbit serum with the same IgG dilution as that of the primary antibody, showed no protein band (Fig 2). This result proves that the rabbit anti-human Lin28 antibody used in this study cross-reacted with Lin28 in the stallion testes.

### 3. Lin28 expression in the testes of pre- and post-pubertal stages

**Fig 3:**
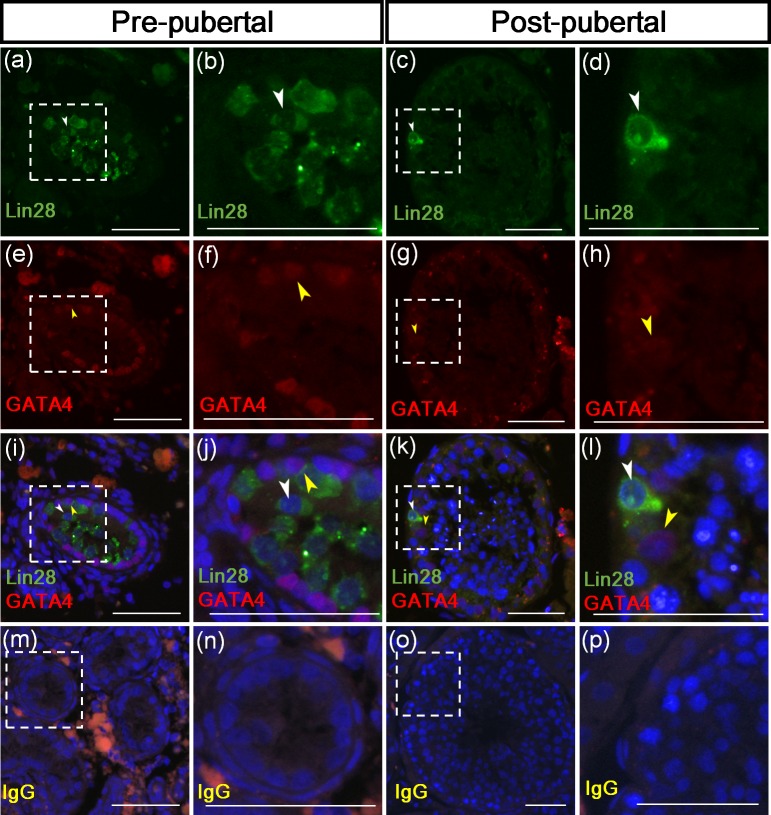
Reproductive stage-dependent localization of Lin28 and GATA4 in stallion germ cells. GATA4 antibody was used as a marker for Sertoli cells. In pre-pubertal stage, the GATA4 positive cells were found located close to the basement membrane of the seminiferous tubules (i and j); howver, in post-pubertal stage, they were located within the germ cell (k and l). The GATA4 positive Sertoli cells showed no co-immunolabeling with Lin28 at both pre- and post-pubertal stages (i, j, k, and l). No immunolabeling of germ cells was observed in the negative control tissue (m, n, o, and p). The white arrow head indicates a Lin28-positive germ cell. The yellow arrow head indicates a GATA4-positive Sertoli cell. Size bar = 200μm.

Immunofluorescence was performed to observe the expression pattern of Lin28 in the testicular tissues of stallions. The reproductive stage-dependent Lin28 positive-germ cells were investigated at both pre- and post-pubertal stages. As hypothesized, Lin28 immunolabeling was exclusively localized in the cytoplasmic areas of germ cells (spermatogonia) in both reproductive stages (Fig 3A–3D and 3I–3L). In the pre-pubertal stage, Lin28-positive germ cells were localized distal from the basal compartment of the seminiferous tubules as observed in the cross-section of a seminiferous tubule (Fig 3A, 3B, 3I and 3J). However, in the post-pubertal stages, germ cells expressing Lin28 were located adjacent to the basement membrane of the seminiferous tubules (Fig 3C, 3D, 3K and 3L). In both reproductive stages, the immunolabeling of Lin28 was localized within the cytoplasmic areas of germ cells (Fig 3A–3D and 3I–3L).

Co-immunofluorescence with Lin28 and GATA4 antibodies was performed to verify Lin28 is not expressed in the Sertoli cells. The GATA4 has been used as a molecular marker for Sertoli cells [4, 11, 12]. As expected, GATA4 positive nucleus of Sertoli cells were found adjacent to the basement membrane of seminiferous tubules in the pre-pubertal testes (Fig 3E, 3F, 3I and 3J), but they were located between germ cells in the post-pubertal stages (Fig 3G, 3H, 3K and 3L). The GATA4 positive Sertoli cells were not co-immunolabeled with Lin28 at both pre-pubertal and post-pubertal stages (Fig 3I–3L). To compare the quantity of Lin28 positive germ cell population in between pre- and post-pubertal stages, the number of Lin28 positive germ cells per 1000 Sertoli cells was counted. The population of Lin28 positive germ cells per 1000 Sertoli cells were 253 ± 8.66 and 29.67 ± 2.18 in pre-pubertal and post-pubertal stages, respectively, and these values were significantly different (p<0.05, Table 1).

**Table 1:** The Lin28 positive germ cell population per 1000 Sertoli cells at different reproductive stages in stallions.

Cell population	Pre-pubertal (n = 3)	Post-pubertal (n = 3)
Lin 28 positive cell population (per 1000 Sertoli cells)	253 ± 8.66 ^a^	29.67 ± 2.18 ^b^

Means with different superscripts indicate significant differences (P < 0.05).

### 4. Reproductive stage dependent co-localization of Lin28 and DAZL in stallion germ cells

**Fig 4:**
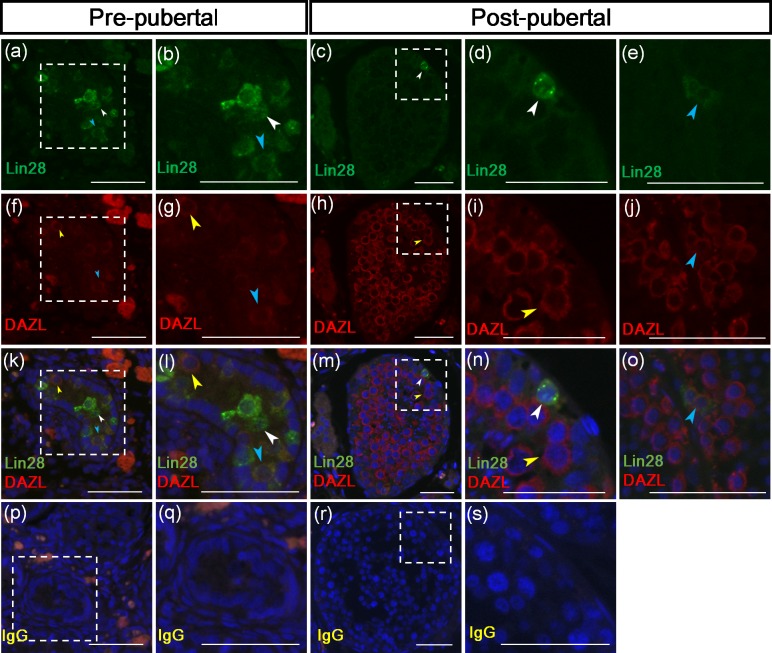
Reproductive stage dependent co-localization of Lin28 and DAZL in stallion germ cells. Three types of staining patterns such as Lin28 only, DAZL only, both Lin28 and DAZL were observed in both reproductive stages (k, l, m, n, and o). Lin 28 only positive germ cells and DAZL only positive germ cells were observed in post-pubertal stage (m and n). Germ cells labeled with both Lin28 and DAZL was also observed (o). No immunolabeling of Lin28 or DAZL was shown on tissues treated with rabbit igG instead of primary antibodies (p, q, r, and s). The white arrow head indicates germ cells stained with Lin28 only. The yellow arrow head indicates a germ cell stained with DAZL only. The blue arrow head indicates a germ cell which was co-immunolabeled with Lin28 and DAZL. Size bar = 200μm.

The DAZL has widely been used as a germ cell marker in various species [13–16]. In our laboratory, DAZL has been demonstrated as a marker for differentiated spermatogonia and primary spermatocytes at the post-pubertal stage [12]. To examine the subpopulations of DAZL positive germ cells, co-staining of Lin28 with DAZL was performed for testicular tissue of pre-pubertal and post-pubertal stallions. At the pre-pubertal stage, the result showed three types of co-immunolabeling patters including spermatogonia immunolabeled with Lin28 only, DAZL only, or both Lin28 and DAZL (Fig 4K and 4L). The rates of spermatogonia immunolabeled with Lin28 only, DAZL only, or both Lin28 and DAZL out of 2000 spermatogonia were 81.78 ± 8.49, 7.4 ± 4.38, and 10.82 ± 4.21%, respectively in pre-pubertal stages (Table 2). In post-pubertal stages, unlike pre-pubertal stages, most spermatogonia and primary spermatocyte were immunolabeled with DAZL (99.08 ± 0.43%) as demonstrated previously [12], whereas spermatogonia immunolabeled with Lin28 only was rarely detected (Fig 4M–4O). Two types of co-immunolabeling patters including spermatogonia immunolabeled with Lin28 only (0.53 ± 0.19%) or both Lin28 and DAZL (0.38 ± 0.26%) were detected (Fig 4M–4O). Tissues for negative control treated with rabbit IgG instead of primary antibody showed no immunolabeling germ cell (Fig 4P–4S).

**Table 2:** The rate of Lin28 and/or DAZL positive germ cell population per 2000 spermatogonia at different reproductive stages in stallions.

Immunolabeling	Pre-pubertal (n = 3)	Post-pubertal (n = 3)
Lin28 only	81.78 ± 8.49^a^	0.53 ± 0.19^b^
DAZL only	7.4 ± 4.38^a^	99.08 ± 0.43^b^
Lin28 and DAZL	10.82 ± 4.21^a^	0.38 ± 0.26^b^

Means with different superscripts indicate significant differences (P < 0.05).

### 5. Clonal arrangement of Lin28 positive spermatogonia in the seminiferous tubules

**Fig 5:**
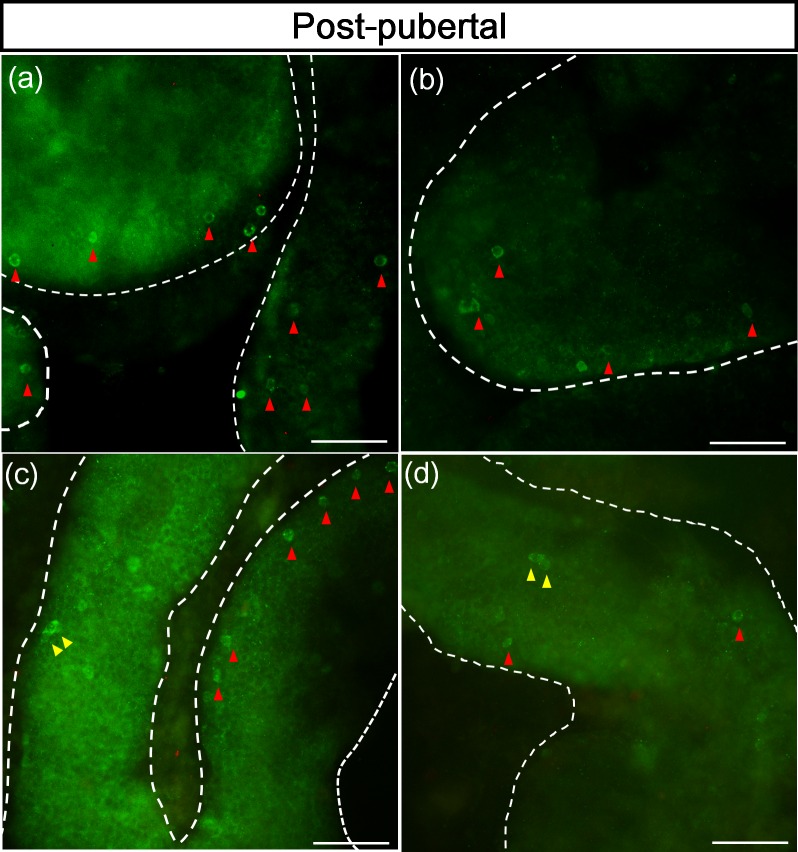
Clonal arrangement of Lin28 positive spermatogonia in the seminiferous tubules. Whole mount staining of seminiferous tubules was performed to find out the size of Lin28 positive spermatogonia subpopulations. Lin28 positive cells were spermatogonia A_s_, (blue arrow head) and A_pr_ (yellow arrow head). Most of the Lin28 positive cells which were observed on the whole mount staining result of this study was a A_s_ spermatogonia, Size bar = 200μm.

Whole mount staining of seminiferous tubules was utilized to find out the size of the spermatogonia subpopulations expressing Lin28. The seminiferous tubules of post-pubertal stallions were used for whole mount staining because low population of Lin28 positive germ cells is necessary to identify the actual size of spermatogonia colony. The result of whole mount staining showed that the Lin28 expression was detected in the cytoplasm of germ cells. The Lin28 positive cells were found as A_s_ and A_pr_ spermatogonia (Fig 5A–5D). Most of the Lin28 positive cells which were observed on the whole mount staining result of this study was A_s_ spermatogonia. No longer than A_pr_ spermatogonia was labeled with Lin28 antibody in this study.

### 6. Potential use of Lin28 antibody for immunocytochemistry

**Fig 6:**
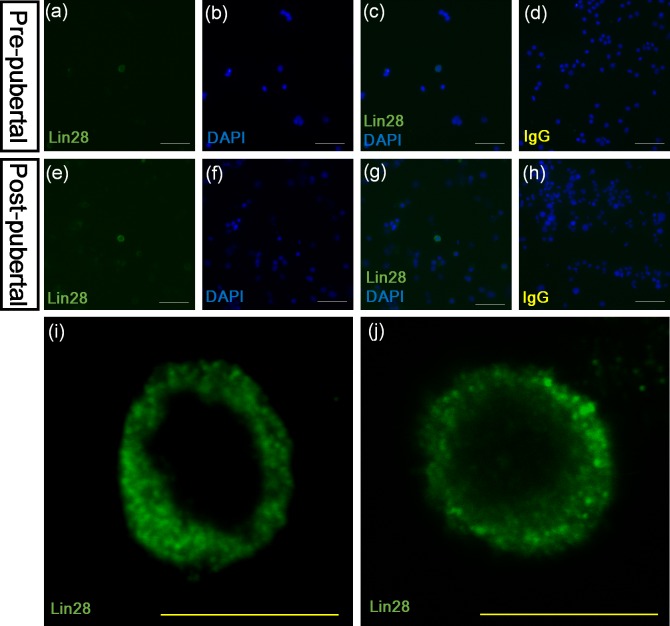
Single germ cells stained with Lin28 antibody using immunocytochemistry. Lin28 antibody staining was also detected in the cytoplasmic area of single germ cells with immunocytochemistry (a, b, c, e, f, and g). No staining was appeared on the tissue treated with rabbit serum (d, and h). Observation of the single germ cells using a confocal microscopy demonstrated that Lin28 antibody was expressed in the cytoplasmic areas of single germ cells (i and j). White size bar = 200 μm, Yellow size bar = 40 μm.

In our laboratory, immunocytochemistry was performed with Lin28 to investigate possible use of the Lin28 antibody in *in vitro* studies. The result of immunocytochemistry demonstrated that the single germ cells were immunolabeled with Lin28 (Fig 6A–6H). Observation of immunolabeling pattern of Lin28 using the confocal microscope showed the cytoplasmic staining of Lin28 in the single cell (Fig 6I and 6J).

## Discussion

This study was performed to investigate the expression patterns of Lin28 in stallion testes at different reproductive stages. The result of RT-PCR indicated that Lin28 mRNA was expressed in the testes of stallions. The result of western blot demonstrated that the Lin28 protein was present in the testes of stallions and rabbit anti human Lin28 antibody had cross-reactivity to Lin28 in stallion testes. The result of immunofluorescence demonstrated that Lin28 was expressed in the cytoplasmic area of spermatogonia in both pre-pubertal and post-pubertal stallions. These data are comparable with Lin28 expression in the cytoplasmic area of spermatogonia in mice [8], monkey and human [10]. The results suggest that Lin28 expression pattern in spermatogonia is conservative throughout the species. Several germ cells located in the center of seminiferous tubules were immunolabeled in pre-pubertal stallions. In contrast, in the post-pubertal stallions, Lin28 positive germ cells are rarely observed and they were located adjacent to the basement membrane of seminiferous tubule. The staining patterns of Lin28 are similar with those of UTF1 expression patters in stallions, indicating that these cells are early stage of spermatogonia [4]. Although expression patterns of UTF1 and Lin28 in spermatogonia are similar, subpopulation of spermatogonia positive for Lin28 appears to be smaller than those positive for UTF1. The number of Lin28 positive cells per 1000 Sertoli cells in pre-pubertal stages (29.67 ± 2.18) was less than the number of UTF1 positive cell population (78.42 ± 17.83) reported from the previous study [4]. Similarly, the number of Lin28 positive germ cells per a round cross-section of seminiferous tubule (0.14 ± 0.07) was also lower than that of UTF1 positive germ cell population (1.48 ± 0.24). These evidences prove that Lin28 is expressed within early subpopulation of undifferentiated spermatogonia compared with UTF1 positive germ cells. This speculation is supported by our whole mount staining for seminiferous tubules of post-pubertal stages. The result of whole mount staining with Lin28 demonstrated that spermatogonia A_s_ and A_pr_ were immunolabeled with Lin28. The result of whole mount staining for seminiferous tubules of mice demonstrated that Lin28 was expressed from A_s_ to 32 chains of A_al_ spermatogonia [8]. These results suggest that the subpopulation of spermatogonia expressing Lin28 varies depending on species. Different subpopulation of spermatogonia positive for Lin28 may be because the size of subpopulation of spermatogonia associated with “stemness” is different among species [2, 5].

In this study, the result of co-immunolabeling between Lin28 and DAZL was detected in both reproductive stages of stallions. During pre- and post-pubertal stage, some but not all of Lin28 positive spermatogonia were also immunolabeled with DAZL. The DAZL is an important factor for differentiation of spermatogonia. In the absence of the RNA-binding protein encoded by the DAZL gene, differentiation of spermatogonia A was inhibited [17]. Thus, we speculate that germ cells immunolabeled with Lin28 only may be spermatogonia maintaining undifferentiated stage. Whereas, Lin28 positive germ cells stained with DAZL may be spermatogonia undergoing further differentiated stage. In the present study, immunolabeling of Lin28 was not co-immunolabeled with GATA4 positive Sertoli cells. Also Lin28 is not detectable on the Leydig cells located within the interstitial space. Therefore, Lin28 as a molecular marker can be used to isolate and identify undifferentiated SSCs.

As a pluripotency factor, Lin28 is mainly expressed in pluripotent embryonic stem cells of mice [18] and human [19]. The results of previous studies suggest that Lin28 may play an important role in controlling the pluripotency of ES cells [18–20]. Lin28 is also one of reprogramming factors together with OCT4, SOX2, and NANOG, that can reprogram somatic cells to induced pluripotent stem cells [21]. Several research teams have demonstrated that SSCs can be pluripotent cells. Kanatsu-Shinohara and coworkers [22] showed that neonatal mouse testis derived ES-like cells differentiated into various types of somatic cells. Furthermore, teratomas were produced after injection of this type of cell line into nude mice. Similar result was also reported with SSCs derived from adult mouse [23] and adult human testes [24]. The results of previous study and the rarity of Lin28 positive spermatogonia in the stallion testes led us to hypothesize that Lin28 positive spermatogonia A_s_ or A_pr_ may have pluripotency. However, it is still debating whether SSCs can be pluripotent or not.

In the present study, the function of Lin28 is not investigated. However, much evidence has demonstrated that Lin28 plays a critical role in remaining undifferentiated status of cells. Specifically, the Lin28 inhibits Drosha processing of the pri-let-7s by binding onto the loop region of pri-let7 [25]. In the cytoplasm, Lin28 binds to the pre-let7a stem to prevent Dicer processing, which turns pre-let7 into the mature let7a [26]. Thus, as the level of Lin28 decreases, the conversion from pri-let-7s into their pre-miRNA [7] and from pre-miRNA into the mature let7a can be induced [26]. Thus, low level of Lin28 in the cytoplasmic of spermatogonia cannot inhibit the process of conversion to be the mature let7a, leading for the cellular differentiation. On the other hand, high level of Lin28 results in promoting a less differentiated state [6]. These results suggest that Lin28 may play a role in maintaining undifferentiated status of SSCs in stallion testis. Thus, it can be utilized as a molecular marker for undifferentiated SSCs.

In summary, the present study has demonstrated that Lin28 can be used as a putative marker for early subpopulations of undifferentiated spermatogonia in stallion testes. Also Lin28, with DAZL antibody, is applicable to monitoring undifferentiated spermatogonia for the *in vitro* study of undifferentiated SSCs.

## Supporting Information

S1 Table Horse testicular sample log. (XLSX)

S2 Table Raw data for immunolabeled germ cell counting. (XLSX)
